# Toroidal metasurface resonances in microwave waveguides

**DOI:** 10.1038/s41598-019-44093-7

**Published:** 2019-05-17

**Authors:** Dimitrios C. Zografopoulos, José Francisco Algorri, Antonio Ferraro, Braulio García-Cámara, José Manuel Sánchez-Pena, Romeo Beccherelli

**Affiliations:** 1 Consiglio Nazionale delle Ricerche, Istituto per la Microelettronica e Microsistemi (CNR-IMM), Rome, 00133 Italy; 2 Department of Electronic Technology, Carlos III University of Madrid, Madrid, 28911 Spain

**Keywords:** Electrical and electronic engineering, Applied physics, Microwave photonics, Sub-wavelength optics

## Abstract

We theoretically investigate the possibility to load microwave waveguides with dielectric particle arrays that emulate the properties of infinite, two-dimensional, all-dielectric metasurfaces. First, we study the scattering properties and the electric and magnetic multipole modes of dielectric cuboids and identify the conditions for the excitation of the so-called anapole state. Based on the obtained results, we design metasurfaces composed of a square lattice of dielectric cuboids, which exhibit strong toroidal resonances. Then, three standard microwave waveguide types, namely parallel-plate waveguides, rectangular waveguides, and microstrip lines, loaded with dielectric cuboids are designed, in such a way that they exhibit the same resonant features as the equivalent dielectric metasurface. The analysis shows that parallel-plate and rectangular waveguides can almost perfectly reproduce the metasurface properties at the resonant frequency. The main attributes of such resonances are also observed in the case of a standard impedance-matched microstrip line, which is loaded with only a small number of dielectric particles. The results demonstrate the potential for a novel paradigm in the design of “metasurface-loaded” microwave waveguides, either as functional elements in microwave circuitry, or as a platform for the experimental study of the properties of dielectric metasurfaces.

## Introduction

Research in the scientific field of metasurfaces has been rapidly expanding in the last years, not only because it provides renewed insight in optics, photonics and, in general, electromagnetics, but also thanks to the powerful tools it offers in the engineering of novel compact devices with unprecedented performance in various applications^1–3^. Metasurfaces are thin periodic arrays of subwavelength resonant scatterers, whose interaction is capable of drastically modifying the properties of light, in ways unachievable by bulk materials of the same subwavelength thickness. Although the elements composing a metasurface can be either metallic or dielectric, the latter are constantly gaining more attention as they do not suffer from ohmic losses, while showing a very rich portfolio of exploitable properties, particularly when high-refractive index materials are employed^4^.

By appropriate design of the metasurface periodicity and of the fundamental unit elements, a myriad of devices have been demonstrated for reflection/refraction control and wavefront shaping^5–11^, lensing^12,13^, holography^14^, control of light emission^15,16^ or photoluminescence^17^, polarization control^18,19^ and polarimetry^20^, generation of vortex beams^21,22^, highly-selective filtering^23,24^, antireflection coatings^25^, enhancing nonlinear processes^26–28^ and more, clearly demonstrating the huge potential of dielectric metasurfaces as low-loss, compact, and functional devices. One of the current trends focuses on the study of dielectric metasurfaces with strong toroidal resonances, associated with the so-called anapole state, which stems from the destructive interference between an electric and toroidal dipole mode with opposite phase and identical angular distribution. Apart from providing a means to observe and study strong toroidal excitations^29–32^, which do not manifest in natural materials, such periodic structures can also be directly exploited in numerous applications, e.g. nanolasers^33^, beam steering^34^, cloaking^35^, broadband absorption^36^, ideal magnetic scattering^37^, enhanced nonlinear effects^38,39^ and the design of high-quality factor resonators^40–43^, to name but a few.

The majority of metasurfaces investigated in the literature are designed for the telecom relevant wavelengths in the near infrared spectrum. However, by scaling their periodic geometry, dielectric metasurfaces can operate in any part of the electromagnetic spectrum, as for instance in the rapidly growing THz and sub-THz technology windows or at even lower microwave frequencies, provided that material losses are not overwhelming^44,45^. In this work, we explore an additional path, namely the possibility to “load” standard microwave waveguides with dielectric particle arrays, which reproduce the resonant features of infinite two-dimensional metasurfaces.

In order to demonstrate the validity of the proposed generic approach, we narrow our focus on the case of metasurfaces with strong dipole toroidal resonances. To this end, we first study the scattering properties of individual high-index dielectric cuboids and observe the excitation of anapole states. Then, all-dielectric metasurfaces using the same cuboids as fundamental building blocks are designed. By invoking key properties of boundary conditions in electromagnetic theory and the resemblance of plane waves with certain waveguide modes, we emulate the metasurface configuration as a linear array of dielectric cuboids embedded in three types of microwave waveguides: (i) parallel-plate waveguides (PPW), (ii) rectangular waveguides (RW), and (iii) microstrip lines (MSL).

Theoretical results demonstrate that the resonant toroidal excitation of the reference all-dielectric metasurface also manifests inside the microwave waveguides, even in the case of a 50-Ω matched microstrip line loaded with only five dielectric elements, thus corroborating the validity of the proposed concept. The limitations in each of the three case studies are discussed and, finally, a discussion is provided on possible applications, such as the design of novel functional microwave components or the experimental investigation of the properties of all-dielectric metasurfaces by means of their emulation in integrated microwave circuits.

## Toroidal Resonances in Cuboid Dielectric Particles

### Multipole decomposition and anapole state excitation in single particles

**Figure 1:**
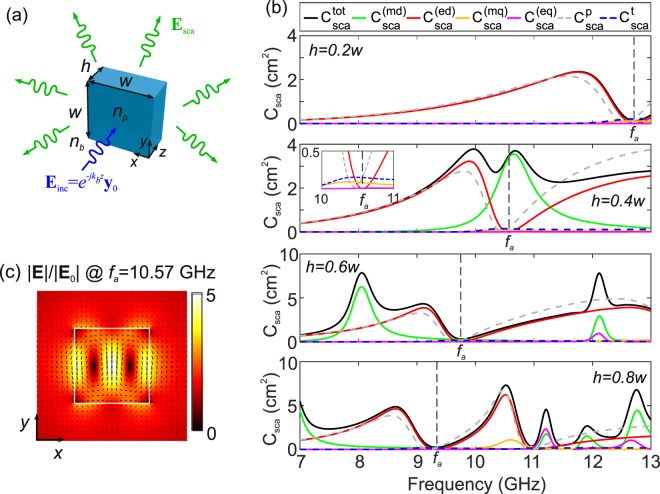
(**a**) Schematic of the dielectric cuboid and definition of the scattering problem. (**b**) Cross-section spectra for the magnetic dipole (md), electric dipole (ed), magnetic quadrupole (mq), electric quadrupole (eq), Cartesian electric (p) and toroidal (t) dipoles for *w* = 8 mm and various values of the thickness *h*. (**c**) Electric field profile at the resonant frequency *f*_*a*_ = 10.57 GHz for *h* = 0.4*w*, where the anapole state is excited.

As a starting point, we investigate the scattering properties of an individual dielectric cuboid with a square cross-section of edge length *w* and thickness *h*, as depicted in Fig. 1. A *y*–polarized planewave impinges perpendicularly on the cuboid and propagates along its short edge, which coincides with the *z*–axis of the system. The electric field **E**(**r**) inside the cuboid is calculated by employing the scattered-field formulation in the finite-element method implemented in the commercial software COMSOL Multiphysics^®^. Then, the multipole decomposition method in Cartesian coordinates is used in order to identify the individual contributions of the dipole and quadrupole electric, magnetic, and toroidal moments to the total scattering cross-section of the dielectric cuboid^46–51^.

In particular, the induced polarization current density **J**(**r**) in the particle, given the *e*^*jωt*^ convention for the harmonic electromagnetic fields, is calculated as

1 $${\bf{J}}({\bf{r}})=j\omega ({\varepsilon }_{p}-{\varepsilon }_{b}){\bf{E}}({\bf{r}}),$$

where *ω* is the angular frequency and *ε*_*p*_ = *ε*_*r*,*p*_*ε*_0_ and *ε*_*b*_ = *ε*_*r*,*b*_*ε*_0_ are the permittivities of the non-magnetic cuboid and surrounding medium, respectively, *ε*_0_ being the vacuum permittivity. The relative permittivities *ε*_*r*,*p*_, *ε*_*r*,*b*_ are equal to the square of the corresponding refractive indices *n*_*p*_, *n*_*b*_.

The dipole moments for the electric, magnetic, and toroidal modes are calculated as

2 $${\bf{p}}=\frac{1}{j\omega }{{\rm{\int }}}_{V}\,{\bf{J}}({\bf{r}})d{\bf{r}}$$

3 $${\bf{m}}=\frac{1}{2{\upsilon }_{b}}{{\rm{\int }}}_{V}\,[{\bf{r}}\times {\bf{J}}({\bf{r}})]d{\bf{r}}$$

4 $${\bf{t}}=\frac{1}{10{\upsilon }_{b}}{{\rm{\int }}}_{V}[({\bf{r}}\cdot {\bf{J}}({\bf{r}})){\bf{r}}-2{r}^{2}{\bf{J}}({\bf{r}})]d{\bf{r}},$$

where *υ*_*b*_ = *c*/*n*_*b*_ is the speed of light in the surrounding medium and the integration is performed in the volume of the cuboid.

The electric, magnetic, and toroidal quadrupole moments are given by

5 $${Q}_{\alpha \beta }^{e}=\frac{1}{j2\omega }{\int }_{V}\,[{r}_{\alpha }{J}_{\beta }+{r}_{\beta }{J}_{\alpha }-\frac{2}{3}{\delta }_{\alpha \beta }({\bf{r}}\cdot {\bf{J}}({\bf{r}}))]d{\bf{r}}$$

6 $${Q}_{\alpha \beta }^{m}=\frac{1}{3{\upsilon }_{b}}{\int }_{V}\,\{{[{\bf{r}}\times {\bf{J}}({\bf{r}})]}_{\alpha }{r}_{\beta }+{[{\bf{r}}\times {\bf{J}}({\bf{r}})]}_{\beta }{r}_{\alpha }\}d{\bf{r}}$$

7 $${Q}_{\alpha \beta }^{T}=\frac{1}{28{\upsilon }_{b}}{\int }_{V}\,[4{r}_{\alpha }{r}_{\beta }({\bf{r}}\cdot {\bf{J}}({\bf{r}}))-5{r}^{2}({r}_{\alpha }{J}_{\beta }+{r}_{\beta }{J}_{\alpha })+2{r}^{2}{\delta }_{\alpha \beta }({\bf{r}}\cdot {\bf{J}}({\bf{r}}))]d{\bf{r}},$$

where the subscripts *α*, *β* = *x*, *y*, *z* and *δ* is the Dirac delta function. In addition, we include the mean-square radii corrections^52^ for the magnetic and toroidal dipole, and the magnetic quadrupole,

8 $${\bar{{\bf{R}}}}_{m}^{2}=\frac{1}{2{\upsilon }_{b}}{\int }_{V}\,[{\bf{r}}\times {\bf{J}}({\bf{r}})]{r}^{2}d{\bf{r}}$$

9 $${\bar{{\bf{R}}}}_{t}^{2}=\frac{1}{28{\upsilon }_{b}}{\int }_{V}\,[3{r}^{2}{\bf{J}}({\bf{r}})-2r({\bf{r}}\cdot {\bf{J}}({\bf{r}}))]{r}^{2}d{\bf{r}}$$

10 $${\bar{{\bf{R}}}}_{\alpha \beta }^{\mathrm{2,}{Q}_{m}}=\frac{1}{42{\upsilon }_{b}}{\int }_{V}\,\{{[{\bf{r}}\times {\bf{J}}({\bf{r}})]}_{\alpha }{r}_{\beta }+{[{\bf{r}}\times {\bf{J}}({\bf{r}})]}_{\beta }{r}_{\alpha }\}{r}^{2}d{\bf{r}}.$$

The mean-square radius corrections for the electric moments are omitted, since such corrections do not contribute to the far-field radiation^46^.

The scattering cross-sections corresponding to each moment are given by

11 $${C}_{{\rm{sca}}}^{({\rm{ed}})}=\frac{{k}_{b}^{4}}{6\pi {\varepsilon }_{b}^{2}}{|{\bf{p}}-j{k}_{b}({\bf{t}}+\frac{{k}_{b}^{2}}{10}{\bar{{\bf{R}}}}_{t}^{2})|}^{2}$$

12 $${C}_{{\rm{sca}}}^{({\rm{md}})}=\frac{{k}_{b}^{4}}{6\pi {\varepsilon }_{b}^{2}}{|{\bf{m}}-\frac{{k}_{b}^{2}}{10}{\bar{{\bf{R}}}}_{m}^{2}|}^{2}$$

13 $${C}_{{\rm{sca}}}^{({\rm{eq}})}=\frac{{k}_{b}^{6}}{20\pi {\varepsilon }_{b}^{2}}\sum _{\alpha \beta }\,{|{Q}_{\alpha \beta }^{e}-j\frac{{k}_{b}}{3}{Q}_{\alpha \beta }^{T}|}^{2}$$

14 $${C}_{{\rm{sca}}}^{({\rm{mq}})}=\frac{{k}_{b}^{6}}{80\pi {\varepsilon }_{b}^{2}}\sum _{\alpha \beta }\,{|{Q}_{\alpha \beta }^{m}-{{k}_{b}}^{2}{\bar{{\bf{R}}}}_{\alpha \beta }^{\mathrm{2,}{Q}_{m}}|}^{2}.$$

assuming unit amplitude for the incident planewave electric field (${{\bf{E}}}_{{\rm{inc}}}={e}^{-j{k}_{b}z}{\bf{y}}$), where *k*_*b*_ = 2*πn*_*b*_/*λ* is the wavevector in the surrounding medium, *λ* being the free-space wavelength. The superscripts (ed), (md), (eq), (mq) denote the electric dipole, magnetic dipole, electric quadrupole, and magnetic quadrupole, respectively.

In the context of identifying and investigating anapole states, it is convenient to define the magnitudes ${C}_{{\rm{sca}}}^{{\rm{p}}}$ and ${C}_{{\rm{sca}}}^{{\rm{t}}}$

15 $${C}_{{\rm{sca}}}^{{\rm{p}}}=\frac{{k}_{b}^{4}}{6\pi {\varepsilon }_{b}^{2}}{|{\bf{p}}|}^{2}$$

16 $${C}_{{\rm{sca}}}^{{\rm{t}}}=\frac{{k}_{b}^{6}}{6\pi {\varepsilon }_{b}^{2}}{|{\bf{t}}+\frac{{k}_{b}^{2}}{10}{\bar{{\bf{R}}}}_{t}^{2}|}^{2},$$

which correspond to the cross-sections of the Cartesian electric and toroidal dipoles, respectively. These two dipoles may interfere constructively or destructively and their combined contribution to the total electric dipole scattering cross-section is given in Equation (11).

Neglecting the contribution of orders higher than the quadrupoles, the scattering cross-section of the cuboid is approximated as

17 $${C}_{{\rm{sca}}}={C}_{{\rm{sca}}}^{(\mathrm{ed})}+{C}_{{\rm{sca}}}^{({\rm{md}})}+{C}_{{\rm{sca}}}^{({\rm{eq}})}+{C}_{{\rm{sca}}}^{({\rm{mq}})}\simeq {C}_{{\rm{sca}}}^{{\rm{tot}}}.$$

The exact total scattering cross-section ${C}_{{\rm{sca}}}^{{\rm{tot}}}$ of the particle is calculated by integrating the outgoing power flow over the particle’s surface, normalized to the energy flux of the incident wave *P*_in_

18 $${C}_{{\rm{sca}}}^{{\rm{tot}}}=\frac{1}{{P}_{{\rm{in}}}}{\int }_{S}\,{\bf{n}}\cdot {{\bf{P}}}_{{\rm{sca}}}dS,$$

where **P**_sca_ is the Poynting vector of the scattered field and *P*_in_ = 0.5*n*_*b*_/*Z*_0_, *Z*_0_ being the characteristic impedance of vacuum.

Following the described procedure, we investigate the scattering properties of a dielectric cuboid with *w* = 8 mm, embedded in air (*n*_*b*_ = 1), in the microwave spectral range from 7 to 13 GHz, which includes the highly relevant X-band (8–12 GHz). The relative permittivity of the cuboids is *ε*_*r*,*p*_ = 24.5(1 − *j*4 × 10^−5^) and it corresponds to state-of-the-art low-loss microwave materials used for the fabrication of dielectric resonators in the X-band^53^. The thickness *h* of the cuboid is varied from *h* = 0.2*w* to *h* = 0.8*w* in four steps and the scattering cross-sections corresponding to the various multipole moments, as well as the total cross-sections, are calculated. The results are shown in Fig. 1(b). As the thickness increases and the cuboid becomes electrically denser, higher-order moments appear in the high-frequency edge of the spectral window. In principle, by controlling the cuboid dimensions it is possible to tune the contributions of the various moments at a given frequency.

In this study, we focus on the conditions for the excitation of the so-called anapole state, identified by a crossover between the Cartesian electric ${C}_{{\rm{sca}}}^{{\rm{p}}}$ and toroidal ${C}_{{\rm{sca}}}^{{\rm{t}}}$ dipole cross-sections that interfere destructively and lead to a minimum of the total electric dipole cross-section ${C}_{{\rm{sca}}}^{({\rm{eq}})}$ at the frequency marked as *f*_*a*_. For instance, in the case of *h* = 0.4*w* this condition is fulfilled at the frequency *f*_*a*_ = 10.57 GHz, as evidenced in the inset of Fig. 1(b). The profile of the electric near-field, shown in Fig. 1(c), is calculated on the *x*−*y* cross-section at the middle of the cuboid (*z* = *h*/2) and it exhibits a double loop of opposite circular displacement currents, characteristic of the anapole state^54^. It is pointed out that although the anapole state leads to zero far-field radiation for the electric dipole moment, the total scattering cross-section is generally not zero, as there are contributions from other multipole moments. In particular, in the case here discussed (*h* = 0.4*w*), the total scattering at the anapole frequency is dominated by the magnetic dipole moment.

### Toroidal resonances in dielectric metasurfaces

**Figure 2:**
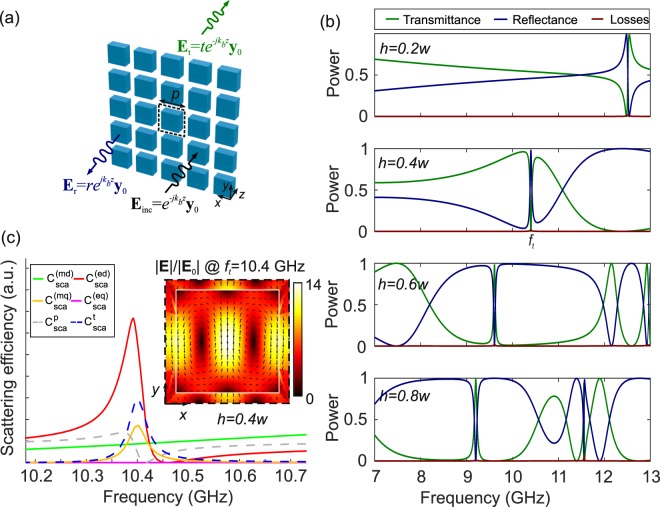
(**a**) Schematic of a metasurface array with pitch *p*, composed of the dielectric cuboid defined in Fig. 1 and definition of the planewave transmission problem. (**b**) Power transmittance, reflectance, and losses spectra of the metasurface for various values of cuboid thickness *h* for *w* = 8 mm and *p* = 9.6 mm. (**c**) Scattering efficiency of the various multipole modes for the metasurface with *h* = 0.4*w* and the electric field profile of the unitary cell at the resonant frequency *f*_*t*_ = 10.4 GHz, where the toroidal dipole mode is excited.

Next, we study the electromagnetic response of metasurfaces consisting of a square periodic lattice, with pitch *p*, of dielectric cuboids in air (*n*_*b*_ = 1), as shown in Fig. 2(a). A *y*–polarized planewave impinges perpendicularly on the metasurface and propagates along the *z*–axis of the structure, as defined in Fig. 2(a). A single unit cell of the metasurface is simulated, by applying periodic boundary conditions (PBC) at the *x*–*z* and *y*–*z* planes and the metasurface spectral response, namely power transmittance (*T* = |*t*|^2^) and reflectance (*R* = |*r*|^2^) is recorded. In all cases examined, the lattice pitch is less than half-wavelength with respect to the high-frequency end of the spectral window (13 GHz), hence no diffraction occurs.

In order to provide an analogy with the study of the scattering from the individual cuboid presented in Fig. 1, we investigate the case of cuboids with width *w* = 8 mm and four thickness values, whereas the lattice pitch is fixed at *p* = 1.2*w* = 9.6 mm. Figure 2(b) shows the transmittance, reflectance, and losses (stemming from dielectric absorption in the cuboid) spectra for a metasurface with different thickness. The interference of the moments supported by the cuboids, as well as their collective oscillations mediated by the metasurface geometry lead to resonances of different Fano lineshapes.

As a case study for our investigation, we focus again on the structure with *h* = 0.4*w*, which shows a sharp peak (dip) in reflectance (transmittance) embedded in a slowly varying spectral background. This resonance is observed at 10.4 GHz, which is very close to the anapole frequency of the corresponding individual cuboid studied in Fig. 1. In order to elucidate the origin of the observed resonance, we calculate the relative scattering efficiency of the various multipole modes, using the multipole analysis described previously, where the electric field **E**(**r**) is calculated in a unit cell of the metasurface. The results of this analysis are plotted in Fig. 2(c), revealing a very strong electric dipole contribution, which stems mainly from the excitation of the toroidal dipole mode, as further corroborated by the profile of the electric field shown in the inset. The contribution of the magnetic quadrupole moment is also noticeable, while all other moments are practically suppressed.

Since toroidal modes are hard to observe in natural materials, both metallic and dielectric metasurfaces have been recently proposed as a platform to investigate and harness the properties of this particular class of electromagnetic modes^29–32,55–57^. Here, we focus on the example of a dielectric metasurface and in the following section we show how it is possible to observe a similar behaviour inside microwave waveguides loaded with dielectric materials in electromagnetically similar configurations that retain the main properties of the equivalent 2-D infinite metasurfaces.

## Microwave Waveguides Loaded with Toroidal Resonators

### Parallel-plate waveguide

**Figure 3:**
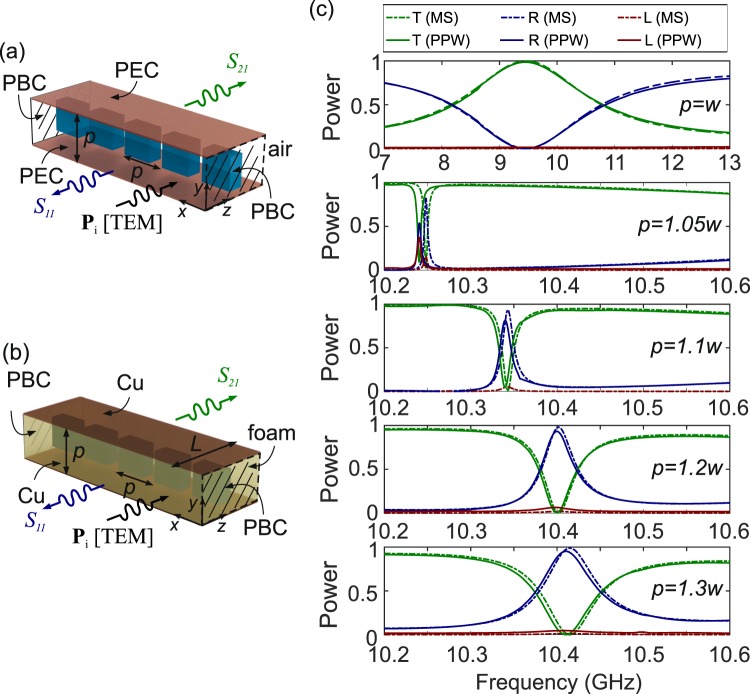
(**a**) Schematic of an ideal parallel-plate waveguide (PPW) formed between two PEC sheets and loaded with a linear array of cuboids. The waveguide height is equal to the array pitch *p*. (**b**) Schematic of a realistic PPW with the same geometrical dimensions, but formed between two Cu sheets and filled with a low-permittivity, low-loss foam. (**c**) Power transmittance, reflectance, and loss spectra of both the ideal metasurface (MS) and the realistic PPW for different values of the pitch *p*, for *w* = 8 mm and *h* = 0.4*w* = 3.2 mm.

According to electromagnetic theory, periodic boundary conditions are equivalent to perfect electric conductor (PEC) conditions, provided the electric field is polarized perpendicularly to the boundary surface. This implies that under such conditions the periodicity of the 2-D metasurface can drop by one order, by employing the structure shown in Fig. 3(a). The square lattice of cuboids is reduced to a 1D problem, i.e., a periodic linear array placed between two PEC sheets separated by a distance equal to the metasurface pitch *p*. This configuration is essentially a parallel-plate waveguide (PPW), which supports a fundamental TEM mode without cutoff, namely at all frequencies. The profile of the TEM mode matches the planewave excitation of the 2-D metasurface. A similar approach was implemented recently for the theoretical demonstration of plasmonic cloaking inside metal-insulator-metal plasmonic integrated waveguides^58^. In a similar context, the electromagnetic equivalence of the TE_10_ RW and TE_1_ PPW modes was exploited to reduce the dispersion in a X-band microwave RW, by loading it with an array of conducting dipoles^59^.

A realistic approach for the PPW is shown in Fig. 3(b). The PEC sheets are substituted by copper with finite conductivity *σ* = 5.81 × 10^7^ S/m and the waveguide is filled with a commercially available, very low-dielectric constant polymethacrylimide foam characterized by *ε*_*r*,*b*_ = 1.046(1 − *j*0.0017)^60^, which emulates the air background medium and provides both mechanical stability and the template in which the dielectric cuboids are embedded. The structure is investigated by defining two ports for the TEM mode. The input Port #1 is excited and the S-parameters of the system *S*_11_ and *S*_21_ are simulated for a waveguide length of *L* = 40 mm. The power transmittance and reflectance of the PPW are calculated as *T* = |*S*_21_|^2^ and as *R* = |*S*_11_|^2^, respectively.

Figure 3(c) presents the PPW transmittance and reflectance spectra for cuboids of *w* = 8 mm and *h* = 3.2 mm for various values of the pitch/PPW height. The results are directly compared to the corresponding spectra of the equivalent 2D metasurfaces. In the case *p* = *w*, the cuboid array degenerates into a dielectric slab of thickness *h*, leading to a Fabry-Pérot response that provides the spectral background in which the multipole modes manifest, as discussed in the case of the results of Fig. 2(b). In all other cases (*p* ≠ *w*), the toroidal resonance is observed. Shorter pitch values lead to higher field enhancement in both the cuboid volume and the spacing between the cuboids and the Cu walls (cf. the electric field profile in the inset of Fig. 2(c)). As a result, the observed resonance is sharper, but also exhibits higher losses. In the case of the 2D metasurfaces, losses stem from absorption in the cuboid, whereas in the case of the PPW they are higher due to the additional conduction losses at the Cu metallic surfaces and absorption in the foam material. As the pitch increases, the field enhancement at the Cu surface is reduced and thus conduction losses are relaxed. The response of the PPW converges to that of the equivalent 2-D metasurface, as evidenced in Fig. 3(c) for the cases *p* = 1.2*w* and *p* = 1.3*w*, which confirms the validity of the proposed approach.

### Rectangular waveguide

Parallel-plate waveguides represent an idealized solution, in the sense that they extend infinitely along one axis. In a practical realization the PPW of Fig. 3(b) has to be terminated at the lateral walls (*y*–*z* planes). Mathematically, a perfect magnetic conductor (PMC) boundary condition would preserve the symmetries of the structure and the obtainable results. However, PMC materials are not found in nature, although there exist practical workarounds, based on elaborate electromagnetic designs^61–63^.

**Figure 4:**
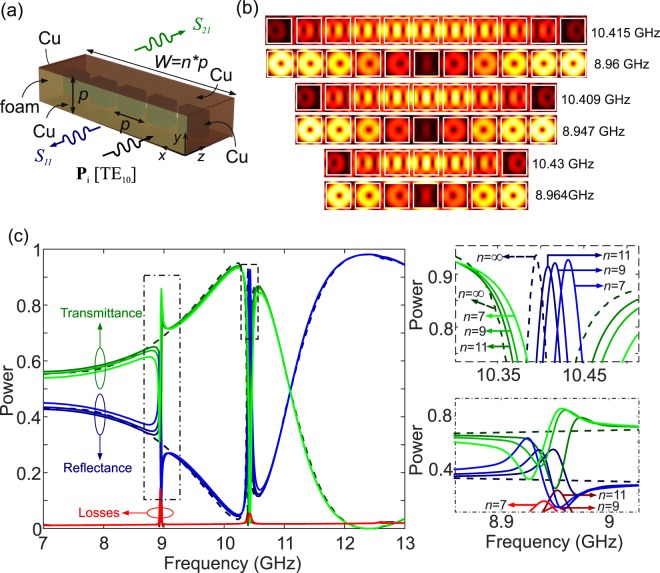
(**a**) Schematic of a microwave rectangular waveguide loaded with *n* cuboids separated by a distance *p*, for a total width of *W* = *np*. (**b**) Electric field profiles (a.u.) at the two resonant frequencies in the spectrum under study for *n* = 7, 9, and 11 cuboids (*w* = 8 mm, *h* = 3.2 mm, *p* = 9.6 mm). (**c**) Power transmittance, reflectance, and losses spectra of the waveguide.

The most common and straightforward solution is to terminate the waveguide with metallic, and therefore quasi-PEC, walls, thus defining the total waveguide width *W*, as shown in Fig. 4(a). The width *W* is selected such that it accommodates a finite number *n* of unit cells of the equivalent metasurface (*W* = *n* · *p*). The structure corresponds to a conventional microwave rectangular waveguide, loaded with a stripe of *n* cuboids placed normal to the RW axis. The fundamental TE_10_ mode propagates at frequencies higher than the cutoff frequency *f*_*c*_ = *υ*/2*W*, where *υ* = *c*/*n*_*b*_ and *n*_*b*_ is the refractive index of the waveguide filling material, in this case the polymethacrylimide foam (${n}_{b}\simeq 1.023$).

Standard MW rectangular waveguides are designed to work in the single-mode regime, which corresponds to a frequency range estimated according to the empirical rule 1.25*f*_*c*_ < *f* < 1.89*f*_*c*_. This range is chosen so that the operating frequency is sufficiently higher than *f*_*c*_ to limit losses and dispersion, but also below the cutoff of the next higher order mode. A typical rectangular waveguide operating in the X-band is WR90 (EIA standard), which has a cross-section of 22.86 × 10.16 mm^2^ and is recommended for operation from 8.2 to 12.4 GHz. However, for the purposes of this investigation, such a waveguide corresponds to a width that accommodates only two periods of the cuboid array. Numerical simulations showed that such a small number of particles is not sufficient to emulate the properties of the corresponding PPW.

A suitable platform to experimentally realize RW that comprise a larger number of dielectric cuboids are substrate integrated waveguides (SIW)^64^. The lateral width *W* of the waveguide is usually defined by two series of via holes that short-circuit the upper and lower metallic sheets, although by proper electromagnetic design the same effect can also be obtained by planar processing^65^. The fundamental mode can be in- and out-coupled in compact and low-profile configurations, for example by slot arrays on top of a suitable beam-forming network, as recently demonstrated in the design and fabrication of SIW metasurface-based leaky-wave antennas^66^, or other SIW mode-launchers based on leaky-wave theory^67^.

Figure 4(c) investigates the transmittance of a 40-mm long RW for a lateral width corresponding to 7, 9, and 11 periods of the 2-D metasurface with *w* = 8 mm and *p* = 1.2*w*. The results demonstrate that the RW behaves almost identically to the benchmark PPW, with the exception of a resonant feature at approximately 8.95 GHz. This corresponds to a resonance induced by the quasi-PEC lateral boundary conditions, as evidenced in the electric field profiles shown in Fig. 4(b). This resonance interferes with the slowly varying reflectance/transmittance spectrum of the cuboid array and leads to the sharp Fano profile evidenced in the spectra of Fig. 4(c). Nevertheless, in the remaining of the spectral window under investigation, the response of the RW is almost identical to the reference PPW. Most importantly, the metasurface-induced toroidal resonance at approximately 10.4 GHz still manifests, as evidenced by both the transmittance spectra and the electric field profiles of Fig. 4(b,c). These profiles demonstrate that the toroidal mode is excited in the cuboid array, with a spatially-variable field amplitude along the *x*-axis, which is consistent with the profile of the TE_10_ mode, i.e. maximum at the RW center and zero at the lateral walls.

The results of Fig. 4 demonstrate that it is possible to reproduce the resonant effect of the 2D reference dielectric metasurface inside a microwave RW with a realistic configuration, which was the main objective of this study. The extra resonant feature close to 8.95 GHz is induced by the presence of the Cu lateral walls and, as mentioned, it can be potentially mitigated in more complex waveguide designs incorporating PMC-emulating elements.

### Microstripe line

**Figure 5:**
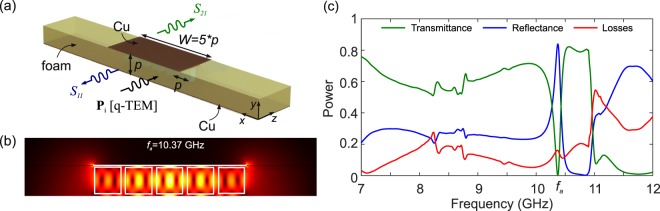
(**a**) Schematic of a microwave microstrip line loaded with *n* = 5 cuboids separated by *p*, for a total stripe width of *W* = 5*p*. (**b**) Electric field profile at the toroidal resonant frequency (*w* = 8 mm, *h* = 3.2 mm, *p* = 9.6 mm). (**c**) Power transmittance, reflectance, and losses spectra of the MMW.

With an eye on practical applications of the proposed concept, in this section we investigate the possibility of loading a standard microwave microstrip line with dielectric cuboids that excite the investigated toroidal modes. The structure of the MSL is shown in Fig. 5(a). The fundamental quasi-TEM mode has an effective permittivity approximated by

19 $${\varepsilon }_{{\rm{eff}}}=\frac{\Re \{{\varepsilon }_{r,b}\}+1}{2}+\frac{\Re \{{\varepsilon }_{r,b}\}-1}{2\sqrt{1+\frac{12p}{W}}}s$$

which for the case of the employed foam leads to ${\varepsilon }_{e{\rm{ff}}}\simeq 1$, as expected. A key characteristic of the MSL is its impedance *Z*_*m*_, which for *W* > *p* is given by the empirical formula^68^

20 $${Z}_{m}=\frac{120\pi }{\sqrt{{\varepsilon }_{{\rm{eff}}}}[\frac{W}{p}+1.393+\frac{2}{3}\,\mathrm{ln}(\frac{W}{p}+1.444)]}\mathrm{.}$$

We investigate the MSL corresponding to the set of values *w* = 8 mm, *p* = 9.6 mm and *W* = 5*p*, which leads to an impedance ${Z}_{m}\simeq 50$ Ω, namely the standard value requested in MW circuits. The MSL is excited by the fundamental, quasi-TEM mode and its S-parameters are recorded for a waveguide length of 40 mm. In the numerical model, perfectly matching layers (PML) are placed at a sufficient distance from the waveguiding region (not shown in Fig. 5(a)). The presence of the dielectric cuboids induces strong scattering of the propagating mode. Contrary to the cases examined before, the field can leak out of the MSL and be absorbed by the PML. Hence, the losses in this case do not only include absorption in the dielectrics or conduction losses at the Cu surfaces but also the electromagnetic energy that couples out of the MSL.

Figure 5(c) shows the spectral response of the investigated MSL. Compared to the reference metasurface spectra of Fig. 2(b), higher losses are clearly observed and the reflectance/transmittance spectra show larger variations. However, when it comes to the main feature, namely the toroidal resonance, it is observed that the toroidal mode is still excited, as corroborated by the electric field profile shown in Fig. 5(b), calculated at the resonant frequency of *f*_*t*_ = 10.37 GHz. Although the quality factor of the resonance is lower, the main properties of the resonance are preserved, namely frequency, spectral shape (a quasi-Lorentzian maximum in reflectance), and field profile. It is stressed that such properties are observed in a 50-Ω, impedance-matched microstrip line, comprising only 5 dielectric cuboids, as compared to an infinite 2D all-dielectric metasurface, which further highlights the potential of the proposed approach in the design of MW components boosted with the properties of dielectric metasurfaces.

## Discussion

In short, we have demonstrated that by properly exploiting analogs in electromagnetic theory it is possible to “load” microwave waveguides with dielectric particle arrays that emulate, to a significant extent, the properties of 2D all-dielectric metasurfaces. This study focused on resonances stemming from the excitation of toroidal modes, but in principle any other metasurface that provides the target spectral response could be employed. For dielectrics with low permittivity dispersion, the design is directly scalable to any frequency, provided the metallic losses are tolerable, as the operating frequency is shifted towards mm-waves and above.

Three standard waveguide types were investigated in order to demonstrate the proof-of-concept. Among these, the PPW provides the best configuration for the demonstration of the toroidal resonance, as it is very similar from the electromagnetics point of view to the reference 2D metasurface. On the other hand, PPWs cannot be easily integrated in a microwave setup. The investigated MSL does not fully reproduce the metasurface spectral response, however it is impedance-matched and therefore it can potentially be embedded as a component in a MSL circuit. Finally, a RW loaded with a moderate number of dielectric elements is proven to be a promising platform for the study of the toroidal resonances in the spectral vicinity of the resonance. Combined with SIW technology, it is a viable approach for the design of “metasurface-loaded” microwave components.

The purpose of designing such “metasurface-loaded” MW waveguides is two-fold; on one hand, novel components could be engineered, exploiting the exotic opportunities offered by dielectric metasurfaces in terms of phase, amplitude, or polarization modulation, which are key properties in any MW circuit. On the other hand, the proposed MW structures can serve as a platform for the investigation of the properties of metasurface arrays, which are more cumbersome to fabricate.

Moreover, dielectrics with very high permittivity values are available at microwave frequencies, which is not the case in the VIS/IR spectrum. High-index dielectric particles lead to a very rich spectrum of resonances, due to the large contrast with the background permittivity^69^. In addition, high-index dielectrics can be employed to shrink the particle size, enabling more compact configurations. In some cases, e.g. ferroelectric materials, such high relative permittivity values are accompanied by tunability, which in principle enables the potential for the design of tunable “metasurface-loaded” microwave components^70^.

Finally, as far as the practical realization of the proposed MW structures is concerned, the availability of high-permittivity materials also relaxes the constrains on the background dielectric. For instance, standard MW substrates, such as PTFE or the Duroid and Ultralam Series by Rogers Corporation, can be used as the template to embed the dielectric particles, which can also come with a properly placed low-loss and lower-index spacer (e.g. alumina)^53^, whose thickness can be adjusted so as to define the periodicity of the particle array. Such an approach would facilitate significantly the fabrication of the loaded waveguides, paving the way for a new class of functional MW components or versatile templates for the study of resonances in arrays of collectively coupled dielectric particles.
