# Author Correction: Toroidal metasurface resonances in microwave waveguides

**DOI:** 10.1038/s41598-020-79342-7

**Published:** 2020-12-21

**Authors:** Dimitrios C. Zografopoulos, José Francisco Algorri, Antonio Ferraro, Braulio García-Cámara, José Manuel Sánchez-Pena, Romeo Beccherelli

**Affiliations:** 1 Consiglio Nazionale delle Ricerche, Istituto per la Microelettronica e Microsistemi (CNR-IMM), 00133 Rome, Italy; 2 Department of Electronic Technology, Carlos III University of Madrid, 28911 Madrid, Spain

Correction to: *Scientific Reports* 10.1038/s41598-019-44093-7, published online 17 May 2019

This Article contains a typographical error in the Acknowledgements section.

“the Ministerio de Economía y Competitividad of Spain (TEC2013-47342-C2-2-R)”.

should read:

"the Ministerio de Economía y Competitividad of Spain (TEC2016-77242-C3-1-R)".

